# Antithyroid drug-induced leukopenia and G-CSF administration: a long-term cohort study

**DOI:** 10.1038/s41598-023-46307-5

**Published:** 2023-11-07

**Authors:** Fumika Kamitani, Yuichi Nishioka, Miyuki Koizumi, Hiroki Nakajima, Yukako Kurematsu, Sadanori Okada, Shinichiro Kubo, Tomoya Myojin, Tatsuya Noda, Tomoaki Imamura, Yutaka Takahashi

**Affiliations:** 1 Department of Diabetes and Endocrinology, Nara Medical University, 840, Shijo-Cho, Kashihara, Nara 634-8522 Japan; 2 Department of Public Health, Health Management and Policy, Nara Medical University, 840, Shijo-Cho, Kashihara, Nara 634-8521 Japan

**Keywords:** Endocrine system and metabolic diseases, Endocrinology

## Abstract

Although antithyroid drug (ATD)-induced agranulocytosis is a significant concern, its risks associated with long-term use and re-administration are not fully elucidated. Therefore, we performed this study to determine the incidence of ATD-induced leukopenia and G-CSF administration using administrative claims database. Retrospective cohort study. This study was performed using the DeSC Japanese administrative claims database. A total of 12,491 patients with newly diagnosed Graves’ disease (GD) who received methimazole or propylthiouracil between April 2014, and February 2021 among 3.44 million patients in the database were included in the study. We measured the six-year incidence of leukopenia and granulocyte colony-stimulating factor (G-CSF) administration. The incidence of leukopenia and G-CSF administration was 1.34% (168 patients) and 0.30% (38 patients), respectively. Leukopenia had a dose-dependent and biphasic incidence. The incidence of leukopenia and G-CSF administration was 37.2 (0.7%) and 8.0 (0.2%) per 1000 person-years during the first 72 days of ATD initiation, whereas it was 3.1 and 0.7 per 1000 person-years during the subsequent 6 years, respectively. The incidence of both outcomes was comparable between first administration and re-administration of ATD. The incidence of ATD-induced leukopenia and G-CSF administration was high in the first 72 days, with a reduced risk for at least 6 years thereafter. The incidence was similar between first administration and re-administration. ATD, a standard therapy, is often administered for a long period; therefore, our findings can guide the treatment of GD.

## Introduction

Antithyroid drugs (ATD) are the standard therapy for Graves’ disease (GD) worldwide. The European Thyroid Association and Japan Thyroid Association guidelines recommend ATD, particularly methimazole (MMI), as the initial therapy for GD. MMI can be used for 12–18 months and discontinued if TSH and TSH receptor antibody (TRAb) levels are within normal ranges. If TRAb levels remain elevated, radioactive iodine (RAI) therapy or surgery is recommended^1^. In selected cases, long-term treatment with ATD is considered because it may increase the chance of remission^2^. In the United States, RAI therapy is the traditional approach, although recently, there is an increasing trend in ATD use and decrease in RAI use^3^.

ATD-induced agranulocytosis is a rare but serious and potentially life-threatening condition. In severe cases, granulocyte colony-stimulating factor (G-CSF) administration is recommended to hasten bone marrow recovery^4,5^. The reported incidence of agranulocytosis is 0.1–0.5%; this condition generally develops within 90 days of therapy initiation^5–8^. The exact incidence of late-stage agranulocytosis remains unclear, despite some reports^7–10^. In addition, these reports include single-center or multicenter studies conducted in specialized hospitals and those from pharmaceutical companies^7^; there is a lack of epidemiological data from general clinical cohort studies with a low risk of bias. Furthermore, the risks of long-term ATD administration and those of ATD re-administration and the incidence G-CSF administration for agranulocytosis are yet unclear^11–14^.

We conducted a long-term retrospective cohort study using data from an administrative claims database in Japan and examined the quantitative risk and characteristics of ATD-associated leukopenia and G-CSF administration.

## Materials and methods

### Study design

The data were provided by DeSC Healthcare Inc. (Tokyo, Japan), and the database comprised claims data of 3.44 million insurance subscribers. The constitution of the database population is reportedly similar to that of the entire population of Japan, and the database can be used for representative medical epidemiology^15^. The claims data included the patients’ personal identifiers, date of birth, sex, date of records, medical diagnoses according to International Classification of Diseases, 10th Revision (ICD-10) codes, medications prescribed, medical procedures, and surgery. These data were anonymously processed when provided. All patient’s data are anonymized with unique ID codes and can be tracked even if patients are transferred to a different hospital, as long as they do not change their health insurance.

### Ethics

This study was conducted in accordance with the Ethical Guidelines for Medical and Health Research Involving Human Subjects established by the Ministry of Health, Labour and Welfare in Japan. This retrospective cohort study was approved by the Ethics Review Committee of Nara Medical University (approval number: 1123-6). The Ethics Committee of Nara Medical University waived the requirement for informed consent because all data were anonymized and deidentifed.

### Outcome

Patients with GD who were insured in the DeSC Healthcare Inc. database and received a new ATD prescription (MMI or propylthiouracil, PTU) between October 2014 and February 2021 were included in the analysis. Patients with unknown age, sex, or observation period were excluded. Patients under 18 years old were excluded because the side effect profiles of antithyroid drugs may differ between children and adults^16^. To evaluate patients newly diagnosed with GD, patients who were prescribed an ATD during the 6 months (washout period; April–September 2014) before the observation period were excluded. To increase the accuracy of the number of G-CSF administrations for ATD-associated agranulocytosis, we first listed all patients who were prescribed G-CSF during the observation period and analyzed the disease names (13,452 patients, Supplementary Table 1), exhibiting that non-Hodgkin lymphoma, lung cancer and breast cancer were the most common. Therefore, GD patients diagnosed with lymphoma /leukemia, lung cancer, or breast cancer (n = 276) were excluded from the analysis. These procedures increased the specificity of ATD-associated G-CSF administration.

GD diagnosis was individuals with a diagnosis code indicating GD and those who were prescribed an ATD (MMI or PTU) at least once during the observation period. The administrative claims data do not include laboratory values such as white blood cell and neutrophil counts. Therefore, leukopenia was defined as presence of a disease code associated with leukopenia or agranulocytosis as per the ICD-10 code^17^ as follows; leukopenia, neutropenia, febrile neutropenia, idiopathic neutropenia, granulocyte depletion, drug-induced granulocytes, agranulocytosis, pancytopenia, and aplastic anemia (Supplementary Table 2) after the initial prescription of an ATD. Additionally, it has been reported that approximately 10% of untreated patients with GD showed a leukocyte count of less than 4000/μL^18^, we excluded patients diagnosed with leukopenia before or on the day of the first ATD administration. To validate the significance of leukopenia, patients who had been administrated G-CSF (filgrastim, lenograstim, or pegfilgrastim) at least once in the ATD-related leukopenia group) were considered to have a severe form of leukopenia. Age was defined as the age when ATD was first prescribed. The observation period for each patient was defined as from the first ATD prescription date to the earliest of the following dates: the date of diagnosis of leukopenia or G-CSF administration, the final ATD prescription date + 90 days, the insurance disqualification date, or the end of the observation period. The observation period for re-administration cases was defined as the number of days since starting the current ATD. For patients who had previously taken both MMI and PTU, the drug (MMI or PTU) administered immediately before the first occurrence of leukopenia or G-CSF administration was defined. According to the guidelines for treating Graves’ disease in Japan, the starting dose of MMI is 15 mg for mild to moderate disease, and for severe disease, it was previously 30 mg but is now MMI 15 mg in combination with potassium iodide^4^. Therefore, we defined MMI≦15 mg as low-dose and MMI > 15 mg as high-dose. When comparing acute onset and late onset of leukopenia and G-CSF administration, the intersection of two linear regression curves with different slopes of cumulative incidence in each Kaplan–Meier curve was determined using an approximation formula. The times of acute and late onset were determined at that inflection point.

### Statistical analysis

**Figure 1:**
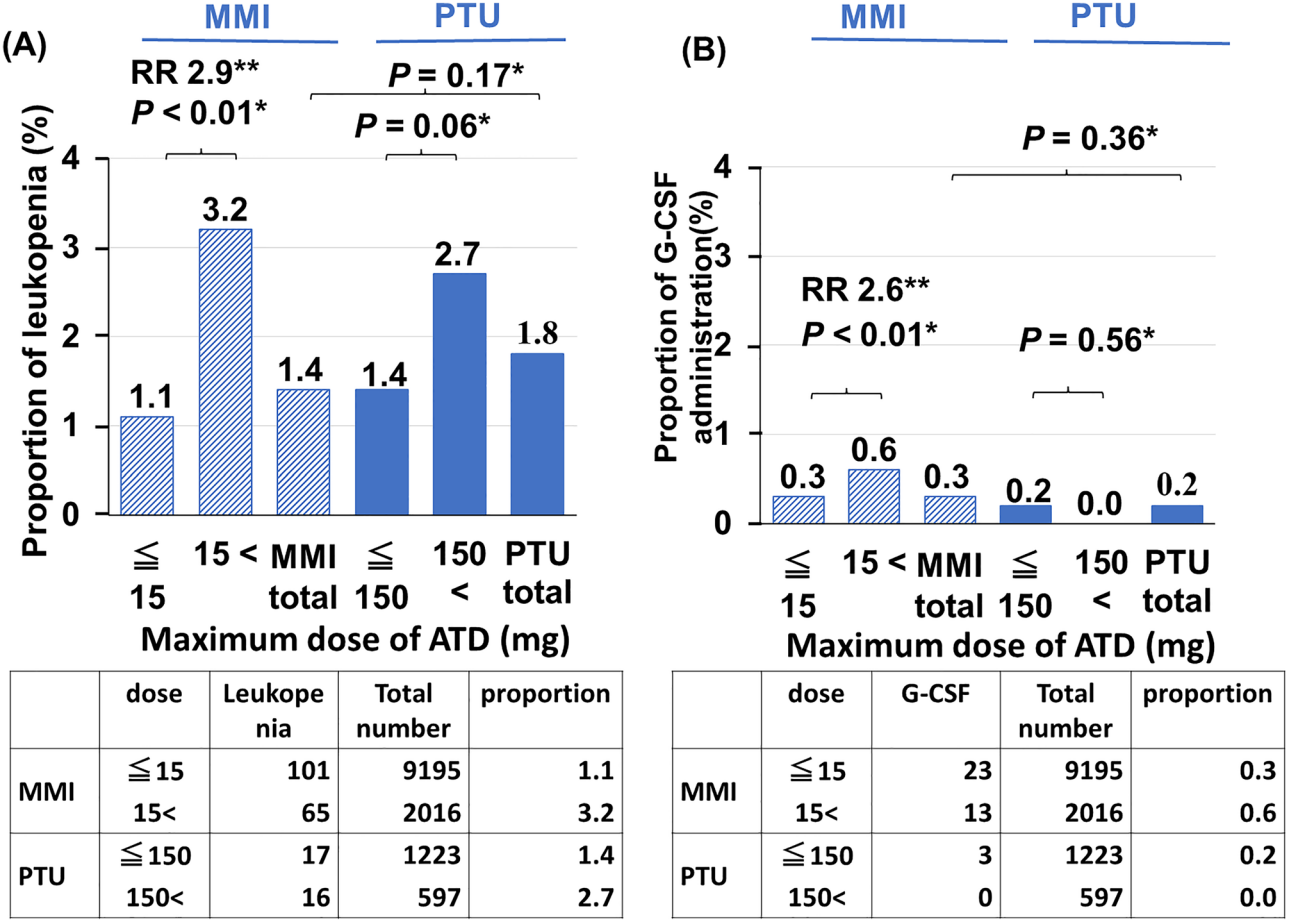
Maximum dose of ATD in patients with leukopenia and G-CSF administration. (**A**) The proportions of patients with leukopenia at the maximum dose of MMI and PTU have been compared. (**B**) The proportion of patients receiving the maximum dose of MMI or PUT who were administered G-CSF have been compared. The proportions of patients with ATD-induced leukopenia were comparable between the MMI and PTU groups (1.4% and 1.8%, respectively. *P* = 0.17). Among patients treated with MMI, the incidence of leukopenia was significantly higher in those receiving high-dose MMI than in those receiving low-dose MMI (RR 2.9, 95%CI 2.1-4.0 *P* < 0.01). The proportions of patients with G-CSF administration were also comparable between the MMI and PTU groups (0.3%, 0.2%. *P* = 0.36). Among patients treated with MMI, the incidence of G-CSF administration was also significantly higher in patients receiving high-dose MMI than in those receiving low-dose MMI (RR 2.6, 95% CI 1.3–5.1, *P* < 0.01). *The Χ^2^ test was performed. **Risk ratios (RRs) have been calculated by dividing the high-dose MMI incidence by the low-dose MMI incidence for leukopenia and G-CSF administration. *ATD* antithyroid drug, *G-CSF* granulocyte colony-stimulating factor, *MMI* methimazole, *PTU* propylthiouracil.

The χ^2^ test was used for statistical analysis of independence and comparison of frequencies for Fig. 1, sex of Tables 1 and 2. When expected values < 5 were included in the table, Fisher’s exact probability test was used instead of the χ^2^ test. The Mann–Whitney U test was used to compare age. The risk ratios (RRs) for the low-dose and high-dose MMI groups and were calculated by dividing the high-dose incidence by the low-dose incidence. The log-rank test was performed to compare the cumulative incidence between the two groups. Missing data were excluded on the basis of the study design. SPSS (IBM SPSS Statistics 28.0.1) was used for all the statistical analyses. Statistical significance was defined as *P* < 0.05.

**Table 1:** Incidence of ATD-induced leukopenia and G-CSF administration.

	Patients receiving an ATD (MMI or PTU)	Patients with leukopenia	Patients without leukopenia	*P*-value	Patients with G-CSF administration	Patients with leukemia without G-CSF administration	*P*-value
Number of patients	12,491	168	12,323		38	130	
Age (years, median) (years, 95% CI)	58 (55.5 to 56.1)	57 (51.8 to 56.4)	58 (55.5 to 56.1)	*P* = 0.20	60 (52.4 to 61.8)	53 (50.5 to 55.9)	*P* = 0.24
Sex (F:M)	(3.0: 1)	(2.7: 1)			(2.5 : 1)	(2.8 : 1)	
Female	9335	123 (1.3%)	9212 (98.7%)	*P* = 0.66	27 (0.3%)	96 (1.0%)	*P* = 0.84
Male	3156	45 (1.4%)	3111 (98.6%)		11 (0.3%)	34 (1.1%)	
Observation period (days, 95% CI)	833 (822 to 844)						

*ATD* antithyroid drug, *G-CSF* granulocyte colony-stimulating factor, *MMI* methimazole, *PTU* propylthiouracil, *CI* confidence interval.

**Table 2:** Comparison of the acute onset and late onset groups.

	Leukopenia acute onset group	Leukopenia late onset group	*P*-value	G-CSF administration acute onset group	G-CSF administration late onset group	*P*-value
Number of patients	88	80	*P* = 0.39	19	19	*P* = 0.35
Person-years	214	2353		232	2111	
Age (years, 95% CI)	53.1 (49.7 to 56.5)	55.1 (51.8 to 58.4)		53.8 (45.1 to 62.5)	60.4 (56.2 to 64.6)	
Sex						
Female	65	58	*P* = 0.86	14	13	*P* = 1.00
Male	23	22		5	6	
Maximum MMI dose						
≦15 mg	46 (52.3%*)	55 (68.8%*)	*P* < 0.01	8 (44.4%*)	15 (83.3%*)	*P* < 0.05
> 15 mg	38 (47.7%*)	18 (31.2%*)		10 (55.6%*)	3 (16.7%*)	
Maximum PTU dose						
≦150 mg	8 (53.3%*)	9 (50.0%*)	*P* = 1.00	1 (100.0%*)	2 (100.0%*)	*P* = 1.00
> 150 mg	7 (46.7%*)	9 (50.0%*)		0 (0.0%*)	0 (0.0%*)	
Days to onset						
Median (days, range)	29.5 (3–72)	422 (74–2073)		43 (9–72)	648 (101–1981)	

*G-CSF* granulocyte colony-stimulating factor, *CI* confidence interval, *MMI* methimazole, *PTU* propylthiouracil.

*Percentage calculated by dividing patients receiving high or low doses of each antithyroid drug by the total number of patients receiving each drug.

## Results

### Incidence of ATD-induced leukopenia and G-CSF administration

In total, 14,751 patients with GD were initially recruited for this study during the observation period. Of these, 1800 patients were not newly diagnosed, the observation period was unknown in 8 patients, 176 patients were under 18 years old, and 276 patients had a diagnosis of lung cancer, breast cancer, lymphoma, or leukemia. Ultimately, the data of 12,491 patients were analyzed (Supplementary Fig. 1). The median observation period was 754 days, approximately 25 months (range, 1–2341 days, 1–78 months).

There were 168 patients with ATD-induced leukopenia (1.3%), 38 of whom were administrated G-CSF (22.6%), during the observation period. The median ages (95%CI) of all patients, patients with leukopenia, patients with G-CSF administration, and patients without G-CSF administration but with leukopenia were 58.0 (55.5–56.1), 57.0 (51.8–56.4), 60.0 (52.4–61.8), and 53.0 (50.5–55.9) years, respectively. Age tended to be older in patients with G-CSF administration than in those without G-CSF administration in patients with leukopenia, although there was no significant difference (*P* = 0.24). The sex ratio (female vs male) was 3.0 vs. 1.0, 2.7 vs. 1.0, 2.5 vs. 1.0, and 2.8 vs. 1.0 for all patients, patients with leukopenia, patients with G-CSF administration, and patients without G-CSF administration but with leukopenia, respectively. There were no significant differences in sex between patients with and without leukopenia (*P* = 0.60), as well as in patients with G-CSF administration and those without G-CSF administration but with leukopenia (*P* = 0.84) (Table 1). The drug names of G-CSF (n = 38) were filgrastim 30 (79.0%), lenograstim 7 (18.4%), and pegfilgrastim 1 (2.6%) (Supplementary Table 3).

### ATD dose at the time of leukopenia and G-CSF administration

Among 168 patients with leukopenia, MMI was prescribed in 153 (91.1%) and the mean dose at the diagnosis of leukopenia was 13.5 mg (95% CI 12.1–15.0 mg). Fifteen patients were treated with PTU, and the mean dose at the time of diagnosis of leukopenia was 150.0 mg (95% CI 99.8–200.2 mg). Of the 38 patients with G-CSF administration, 35 (92.1%) were prescribed MMI, and the mean dose at G-CSF administration was 13.6 mg (95% CI 9.5–17.6 mg). Three patients were treated with PTU, and the mean dose at G-CSF administration was 100.0 mg (95% CI 100–100 mg).

### Maximum ATD dose in patients with leukopenia and G-CSF administration

Of the 12,491 patients, MMI was prescribed in 10,671 (85.4%), PTU was prescribed in 1280 (10.3%), and both MMI and PTU were prescribed in 540 (4.3%) during the observation period. In terms of the dose, 9195 patients (70.5%) were treated with low-dose MMI (≤ 15 mg) and 2016 (15.5%) were treated with high-dose MMI (> 15 mg), with mean doses of 8.7 mg and 28.3 mg, respectively. In the PTU group, 1223 patients (9.4%) were treated with low-dose PTU (≤ 150 mg), and 597 (4.6%) were treated with high-dose PTU (> 150 mg), with mean doses of 91.4 mg and 301.3 mg, respectively. The proportions of patients with ATD-induced leukopenia were comparable between the MMI and PTU groups (1.4% vs. 1.8%. *P* = 0.17) (Fig. 1). In patients with MMI, the incidence of leukopenia was significantly higher in patients receiving high-dose MMI than in those receiving low-dose MMI (RR 2.9, 95% CI 2.1–4.0 *P* < 0.01). In patients with PTU, the incidence of leukopenia tended to be higher in patients receiving high-dose PTU than in those receiving low-dose PTU (*P* = 0.06). The proportions of patients with G-CSF administration were also comparable between the MMI and PTU groups (0.3% vs. 0.2%. *P* = 0.36). In patients with MMI, the incidence of G-CSF administration was also significantly higher in patients receiving high-dose MMI than in those receiving low-dose MMI (RR 2.6, 95% CI 1.3–5.1, *P* < 0.01). Although the incidence of G-CSF administration in patients receiving high-dose PTU was lower than that in patients receiving low-dose PTU, there was no significant difference (*P* = 0.56).

### Cumulative incidence of ATD-induced leukopenia and G-CSF administration for 6 years

**Figure 2:**
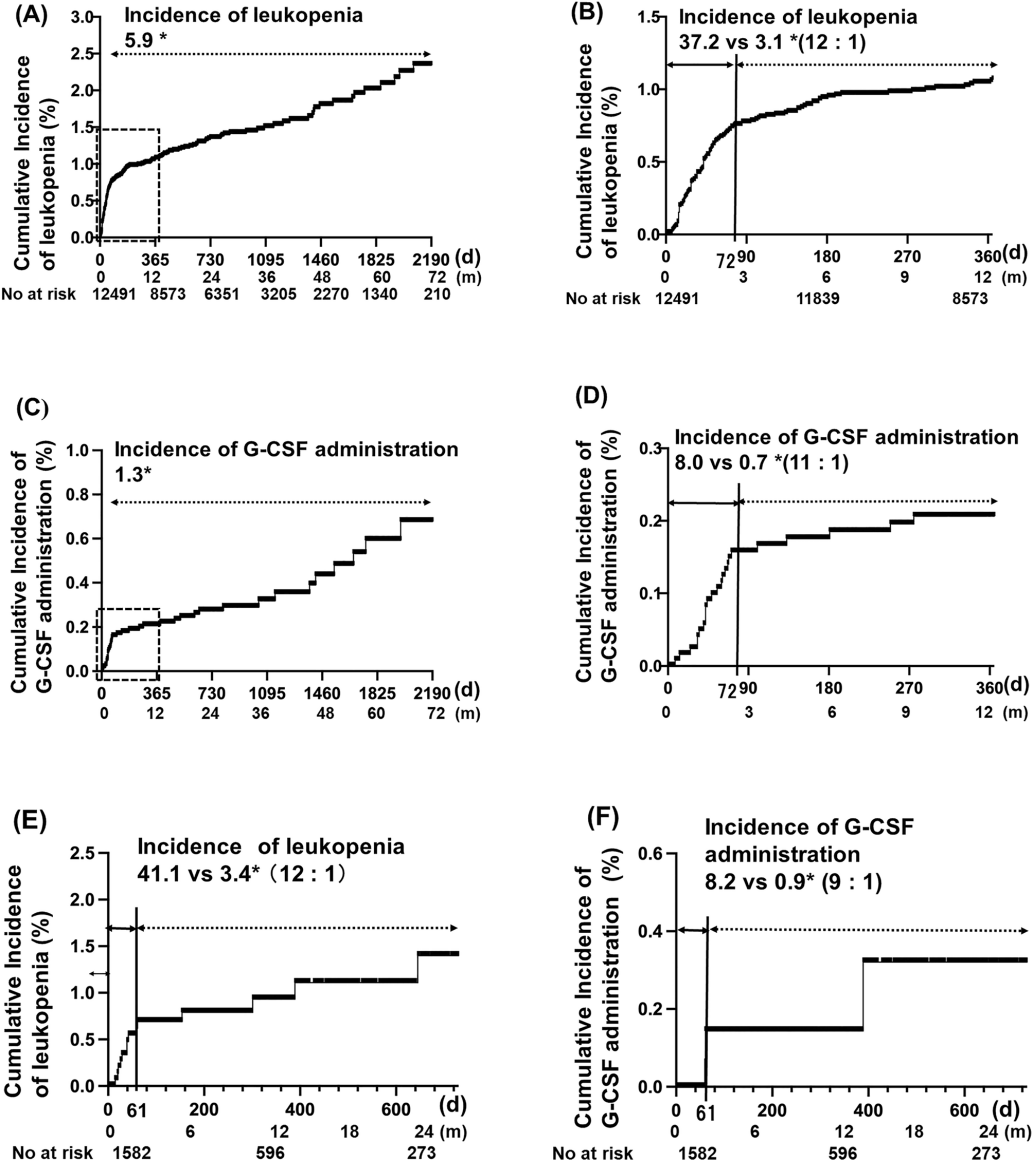
Cumulative incidence of ATD-induced leukopenia and G-CSF administration. (**A**) Kaplan–Meier curve of the cumulative incidence of leukopenia. The total incidence is 5.9 per 1000 person-years. (**B**) A magnified view of a portion of Fig. 2A. Approximation formula for Fig. 2(A) (1) 100y = 0.308x − 7.6, (2) 100y = 0.017x + 13.4, x = 72.2, where x = time [days], y = the incidence [1000 person-years] The acute phase (the first 72 days) and late phase (subsequent days) have been separately analyzed. The incidence of leukopenia in the acute phase is 37.2 per 1000 person-years and that in late phase is 3.1 per 1000 person-years. (**C**) Kaplan–Meier curve of the cumulative incidence of G-CSF administration. The total incidence is 1.3 per 1000 person-years. (**D**) A magnified view of a portion of Fig. 2C. Approximation formula for Fig. 2C (1) 100y = 1.41x − 16, (2) 100y = 0.06x + 82, x = 72.6, where x = time [days], y = the incidence [1000 person-years] The acute phase (the first 72 days) and late phase (subsequent days) have been separately analyzed. The incidence of leukopenia in the acute phase is 8.0 per 1000 person-years and that in late phase is 0.7 per 1000 person-years. (**E**) Kaplan–Meier curve of the cumulative incidence of leukopenia in patients with re-administration of ATD. The acute phase (the first 62th days) and late phase (subsequent days) have been separately analyzed. The incidence of leukopenia in the acute phase is 41.1 per 1000 person-years and that in late phase is 3.4 per 1000 person-years. (**F**) Kaplan–Meier curve of cumulative incidence of G-CSF administration with re-administration of ATD. The acute phase (the first 62 days) and late phase (subsequent days) have been separately analyzed. The incidence of leukopenia in the acute phase is 8.2 per 1000 person-years and that in late phase is 0.9 per 1000 person-years. *Per 1000 person-years. *ATD* antithyroid drug, *G-CSF* granulocyte colony-stimulating factor.

The Kaplan–Meier curve of the time course and the cumulative incidence of leukopenia in the patients with a first ATD administration are shown in Fig. 2A, and B shows a magnified view of a portion of Fig. 2A. The incidence of leukopenia during the entire observation period was 5.9 per 1000 person-years. We determined the intersections of two linear regression curves with different slopes of cumulative incidence in the Kaplan–Meier curves in Fig. 2A and C using an approximation formula, and found the bending point on day 72. Consequently, we defined the acute-onset group as those with onset on or before day 72 and the late-onset group as those with onset thereafter. The incidence in these two groups was then calculated. Interestingly, there were clear differences in the incidence of leukopenia between the first 72 days after ATD initiation (acute onset) and the 6-year follow up period (late onset) (Fig. 2A,B). The incidence of leukopenia in the acute onset group was 37.2 per 1000 person-years (0.7% during the first 72 days), and that in the late onset group was 3.1 per 1000 person-years (Fig. 2B).

The incidence of G-CSF administration in patients diagnosed with leukopenia after ATD treatment during the entire observation period was 1.3 per 1000 person-years (Fig. 2C). Figure 2D shows a magnified view of a portion of Fig. 2C. The same tendency was observed for leukopenia; therefore, we calculated the incidence of leukopenia in the acute and late phases separately (Fig. 2D). The incidence of G-CSF administration for agranulocytosis in the acute onset group was 8.0 per 1000 person years (0.2% during the first 72 days), and that in the late onset group was 0.7 per 1000 person-years (Fig. 2D). These data clearly show that there was more than a tenfold difference in the incidence of leukopenia between the acute onset and late onset groups. Additionally, although the incidence was relatively low, the risk persisted for up to 6 years.

### Comparison of clinical characteristics between the acute- and late-onset groups

The clinical characteristics of patients with ATD-induced leukopenia and G-CSF administration were compared between the acute onset (during the first 72 days) and late onset groups (during the subsequent 6 years), as determined from the Kaplan–Meier curves in Fig. 2A. (Table 2). There were no significant differences in age, sex, and maximum PTU dose between the acute onset and late onset groups. Interestingly, for both leukopenia and G-CSF administration, the acute onset group showed a significantly higher maximum dose of MMI than the late onset group (*P* < 0.01, *P* < 0.05, respectively).

### Comparison of initial MMI dose and MMI dose at G-CSF administration

**Figure 3:**
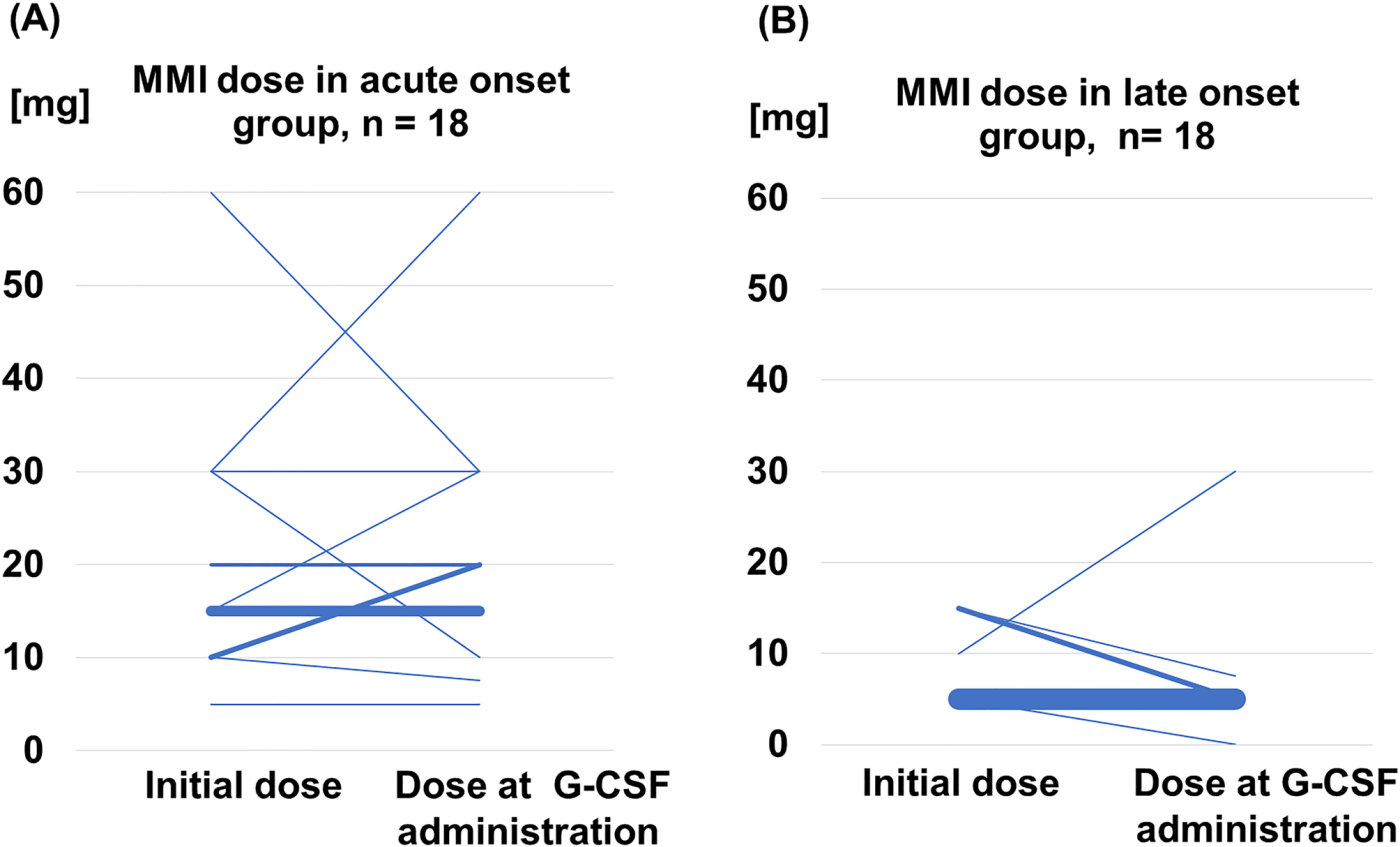
Comparison of the initial MMI dose and MMI dose at G-CSF administration. (**A**) Comparison of the initial MMI dose and MMI dose at G-CSF administration in the acute onset group. Most patients were administered 15 mg of MMI. (**B**) Comparison of the initial MMI dose and MMI dose at G-CSF administration in the late onset group. Most patients were administered 5 mg of MMI.

Comparing the initial MMI dose and the MMI dose of G-CSF administration in patients treated with MMI, most patients in the acute onset group were treated with 15 mg of MMI, and the dose remained unchanged (Fig. 3). Similarly, most patients in the late onset group were treated with 5 mg of MMI, and the dose remained unchanged.

### Cumulative incidence of leukopenia and G-CSF administration with ATD re-administration

We further evaluated the incidence of leukopenia (Fig. 2E) and G-CSF administration (Fig. 2E) in patients who underwent ATD re-administration (Table 3). Among the 12,491 patients treated with ATD, ATD was re-administered in 1528 during the observation period. Among these, 14 exhibited leukopenia and 3 were treated with G-CSF (Table 3). The median discontinuation period of ATD was 299 days (range, 171–1240 days). Among 14 patients, 10 developed leukopenia rapidly during the first 62 days after the initiation of re-administration (Table 3, Cases 1–10, 35 days [range, 16–62 days]), and 4 developed leukopenia slowly during the subsequent days (Table 3, Cases 11–14, 346 days [range, 155–646 days]). Interestingly, the Kaplan–Meier curve of the cumulative incidence of leukopenia and G-CSF administration in patients with re-administration revealed a similar shape to that of the first administration, although the patient number was small in G-CSF administration group (Fig. 2E,F). We calculated the incidence of acute onset (the first 62 days) and late onset (the subsequent days up to 2 years). The incidence of leukopenia in the acute onset group was 41.1 per 1000 person-years (0.7% during the first 62 days), and that in the late onset group was 3.4 per 1000 person-years (Fig. 2E). The incidence of G-CSF administration for leukopenia in the acute onset group was 8.2 per 1000 person-years (0.1% during the 62 days), and that in the late onset group was 0.9 per 1000 person-years (Fig. 2F). Comparison of the Kaplan–Meier curves of the cumulative incidence of leukopenia and G-CSF administration between the first administration and re-administration showed no significant differences (log-rank test, *P* = 0.63, *P* = 0.85, respectively), indicating that the risk of leukopenia and G-CSF administration was similar.

**Table 3:** Clinical characteristics of patients undergoing ATD re-administration.

Case	G-CSF administration	Age (years)	Sex	ATD	ATD course	Comparison with the previous dose of ATD*	Total duration of ATD treatment (days)	Discontinuation period (days)	Re-administration period (days)
1	–	47	M	MMI	2nd	Same	609	321	16
2	–	45	F	MMI	3rd	Decreased	86	296	21
3	–	79	F	MMI/PTU	2nd	Increased	77	462	22
4	–	51	F	MMI	2nd	Increased	515	1240	27
5	–	49	M	MMI	2nd	Decreased	651	186	30
6	–	51	F	MMI	3rd	Decreased	171	231	40
7	–	45	M	MMI	2nd	Same	472	836	40
8	–	53	M	MMI	2nd	Increased	239	421	43
9	G-CSF	26	F	MMI	2nd	Increased	375	178	61
10	G-CSF	25	F	MMI	2nd	Same	152	300	62
11	–	33	F	MMI/PTU	2nd	Increased	537	171	155
12	–	45	M	MMI	2nd	Decreased	702	200	302
13	G-CSF	50	F	MMI	2nd	Same	920	182	390
14	–	35	F	MMI/PTU	2nd	Same	701	576	646

*ATD* antithyroid drug, *G-CSF* granulocyte colony-stimulating factor, *MMI* methimazole, *PTU* propylthiouracil.

*The maximum dose of ATD at leukopenia and/or G-CSF administration was compared with the previous dose.

## Discussion

In this study, we clearly demonstrated that the risk of ATD-induced leukopenia and G-CSF administration was biphasic; its incidence in the acute onset group was 37.2 per 1000 person-years (0.7% during the first 72 days) and that in the late onset group was 3.1 per 1000 person-years, which was 10 times lower than that in acute onset group, with the risk persisting for at least for 6 years. The risk of G-CSF treatment generally administered for management of severe leukopenia in the acute group was 8.0 per 1000 person-years (0.2% during the first 72 days) and that in the late onset group was 0.7 per 1000 person-years. In addition, the risk of leukopenia and G-CSF administration was similar between first ATD administration and ATD re-administration.

ATD treatment for GD is generally provided in outpatient clinics. It is a standard therapy, and ATD has been used to treat GD in more than 90% of patients in Japan^19,20^, 85% of patients in Europe^21^, and more than 40% of patients in the US, where ATD use has been increasing and RAI use has been decreasing^3^. Guidelines recommend ATD, particularly MMI, as the initial therapy for GD, and patients are generally treated with ATD for 12–18 months^1,5,22^. In addition, long-term treatment with low-dose MMI for a total of 60–120 months may also be efficacious, with higher remission rates than those observed with the conventional 18–24 months course^2,23^. Indeed, in real clinical practice, it is not rare to prescribe ATD for a long period; therefore, it is necessary to keep in mind the precise risk of leukopenia and G-CSF administration with long-term treatment. Based on our data, we recommend focusing on this risk in a long term treatment, although the incidence is low.

A previous report has shown that elderly patients are susceptible to agranulocytosis^5^. In this study, we demonstrated that there was a tendency for patients with G-CSF administration to be older. Additionally, there are reports showing that women have a higher risk of developing agranulocytosis^3,5,8^, but there were no significant differences in sex between patients with G-CSF administration and ATD-treated patients in this study.

The reported incidence of agranulocytosis due to MMI and PTU remains controversial; it has been reported that the incidence is significantly higher for PTU than for MMI^24^, although another study found no difference^25^. In this study, there were no significant differences in leukopenia and G-CSF administration between the PTU and MMI groups. However, PTU was prescribed in only 14.6% of the patients in this study, and the relatively small number of patients receiving PTU may influence the results. In terms of the ATD dose, it is reported that the risk of MMI-induced agranulocytosis is higher with an initial dose of 30 mg/d than with a dose of 15 mg/d^26^. In this study, the incidence of leukopenia and G-CSF administration was significantly higher in patients treated with more than 15 mg of MMI than in those treated with 15 mg or less of MMI (RR 2.9 and 2.6,, respectively), supporting previously reported results^26^. The incidence of leukopenia tended to be higher in patients treated with more than 150 mg of PTU than in those treated with 150 mg or less pf PTU (*P* = 0.06). The incidence of G-CSF administration was lower in patients receiving high-dose PTU than in those receiving low-dose PTU. However, this difference was not statistically significant (*P* = 0.56), and the relatively small number of patients receiving high-dose PTU (n = 597) may have influenced the results.

Interestingly, there was a biphasic risk of leukopenia and G-CSF administration during ATD treatment. Although the detailed mechanisms remain unclear, two mechanisms have been proposed: direct ATD toxicity and immune-mediated responses^27–29^. Reactive metabolites produced during ATD oxidization in neutrophils may exert direct toxicity^18^. Autoantibodies against mature blood cells and/or bone precursor cells are involved in immune-mediated responses^29–31^. We postulated that the different incidences of agranulocytosis in the acute and late phases may be explained by these different mechanisms.

There are limited data on the risk of agranulocytosis with ATD re-administration. In a previous study, the clinical characteristics between the first administration and re-administration groups were comparable, including the period after ATD administration and agranulocytosis diagnosis (39.0 days vs. 32.5 days)^32^. In this study, although the patient number of administered G-CSF in the re-administration group was limited; therefore, caution is required when interpreting the findings in G-CSF administration group, but the incidence of leukopenia and G-CSF administration was similar between the first administration and re-administration of ATD. Moreover, we clearly showed that the risk was biphasic in leukopenia group, similar to the first administration. Therefore, it is recommended that similar attention be paid when an ATD is re-administered.

Our study has several limitations and strength. It is important to acknowledge a limitation of this study, notably that due to the characteristics of the administrative claims database, laboratory findings such as thyroid hormone levels and thyroid function index^33^ were not available. Therefore, the appropriateness of antithyroid medication, dosage, and duration of administration cannot be examined. However, since the purpose of this study was to clarify antithyroid drug-related leukopenia and G-CSF administration, we believe that these limitations do not affect the conclusions. Furthermore, agranulocytosis is generally defined as granulocyte count of less than 500/μL; however, the definition of leukopenia in this study cannot exclude the mild leukocytopenia that does not meet the criteria of agranulocytosis. However, we defined not only by leukopenia but also by G-CSF administration, which is generally used for severe leukocytopenia. In addition, patients administered G-CSF for other purposes, such as chemotherapy, were carefully excluded. As a result, the data on leukopenia and G-CSF administration were consistent, suggesting the integrity of this study. Second, we set a wash out period of 6 months for the definition of newly prescribed ATD; therefore, it is difficult to exclude the patients who prescribed ATD more than 6 months before. Third, there is a possibility of underestimating the incidence of leukopenia and G-CSF administration related to ATD because we excluded patients with three malignancies for which G-CSF commonly used, and some of these patients had both ATD and G-CSF treatment. However, the incidence of G-CSF administration with these malignancies was 1.7% (276/16,596); therefore, their influence was negligible and we believe that it did not affect the conclusion. As a strength, this study included a relatively large number of patients with GD who were prescribed ATD, and the observation period was sufficiently long, compared with those of previous studies^6,23^, to enable quantification of long-term risk. Furthermore, the selection bias was small, reflecting real world clinical practice.

In conclusion, the risk of ATD-induced leukopenia and G-CSF administration was biphasic. A high incidence was observed in the first 72 days following the initiation of treatment, and a reduced risk was observed for at least the subsequent 6 years. The incidence was similar in the case of re-administration. ATD is a standard therapy and is often administered for a long period; therefore, our findings can guide treatment for GD.

### Supplementary Information

Supplementary Figure 1.

Supplementary Information.

## Data Availability

The data that support the findings of this study are available from DeSC Healthcare Inc. (Tokyo, Japan) but restrictions apply to the availability of these data, which were used under license for the current study, and so are not publicly available. Data are however available from the corresponding author upon reasonable request and with permission of DeSC Healthcare Inc. (Tokyo, Japan).
