# Supplementary figures and images for: Antithyroid drug-induced leukopenia and G-CSF administration: a long-term cohort study
Source: Sci Rep. 2023 Nov 7;13:19336. doi: 10.1038/s41598-023-46307-5 (PMC10630492; doi:10.1038/s41598-023-46307-5)

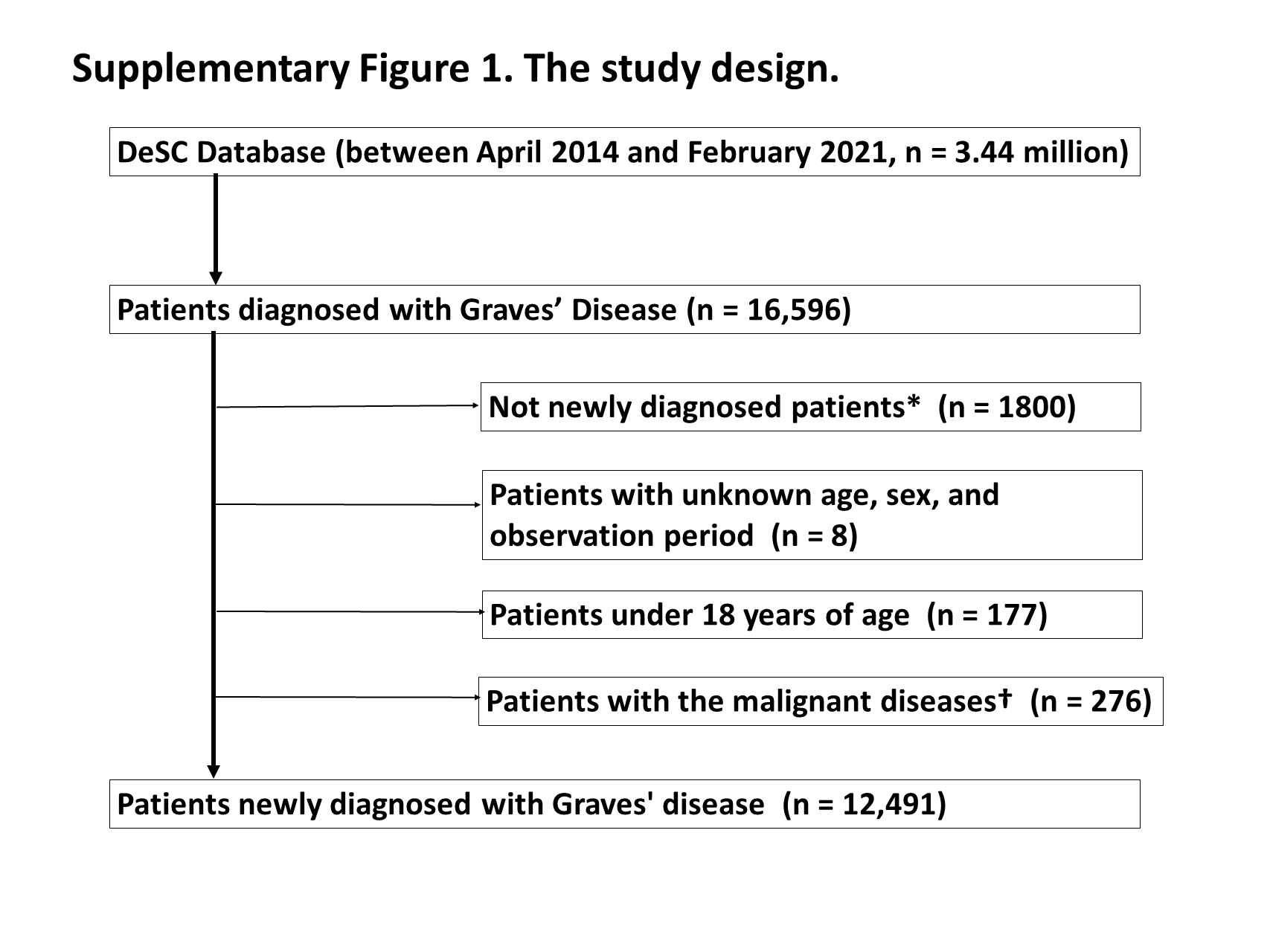

Supplement: Supplementary file 1 — Supplementary Figure 1. [file 41598_2023_46307_MOESM1_ESM.tif]
