# Supplementary material for: Antithyroid drug-induced leukopenia and G-CSF administration: a long-term cohort study
Source: Sci Rep. 2023 Nov 7;13:19336. doi: 10.1038/s41598-023-46307-5 (PMC10630492; doi:10.1038/s41598-023-46307-5)
Supplement: Supplementary file 2 — Supplementary Information. [file 41598_2023_46307_MOESM2_ESM.docx]

**Antithyroid Drug-induced Leukopenia and G-CSF Administration: A Long-term Cohort Study**

Fumika Kamitani^1^, Yuichi Nishioka*^2^, Miyuki Koizumi^1^, Hiroki Nakajima^1^, Yukako Kurematsu^1^, Sadanori Okada^1^, Shinichiro Kubo^2^, Tomoya Myojin^2^, Tatsuya Noda^2^, Tomoaki Imamura^2^, Yutaka Takahashi^1^

**Supplementary Table 1. Number of patients with comorbid malignancy who were treated with G-CSF during the observation period**

| ICD-10 disease names | ICD-10 code | Number of patients |
| --- | --- | --- |
| Other specified and unspecified types of non-Hodgkin lymphoma | C85 | 2507* |
| Malignant neoplasm of the bronchus and lung | C34 | 2483* |
| Malignant neoplasm of the breast | C50 | 2119* |
| Non-follicular lymphoma | C83 | 1464* |
| Malignant neoplasm of the colon | C18 | 1144 |
| Malignant neoplasm of the stomach | C16 | 1090 |
| Malignant neoplasm of the prostate | C61 | 840 |
| Malignant neoplasm of the pancreas | C25 | 642 |
| Myeloid leukemia | C92 | 554* |
| Malignant neoplasm of the ovary | C56 | 542 |
| Malignant neoplasm of the rectum | C20 | 535 |
| Malignant neoplasm of the esophagus | C15 | 515 |
| Lymphoid leukemia | C91 | 487* |
| Multiple myeloma | C90 | 466* |
| Malignant neoplasm of the corpus uteri | C54 | 451 |
| Malignant neoplasm of the bladder | C67 | 422 |
| Follicular lymphoma | C82 | 355* |

G-CSF: Granulocyte colony-stimulating factor; ICD-10, International Classification of Diseases, 10th Revision.

Comorbidity (malignant tumor) in patients prescribed G-CSF during the observation period (n= 13,452). Metastatic tumors and cancers of unknown primary origin were excluded from the analysis. Patients with ICD-10 codes for the three most commonly observed diseases (non-Hodgkin lymphoma, lung cancer and breast cancer) were excluded from the main study. Many of the first-ranked C85 lymphomas were associated with lymphomas with the ICD-10 codes C82–C92; therefore, patients with these diseases and leukemia were also excluded from the analyses.

* Number of patients with ICD-10 disease names indicating these three diseases (lymphoma and leukemia, lung cancer and breast cancer). However, some patients were duplicates.

**Supplementary Table 2. List of disease codes used to diagnose leukopenia**

| Disease codes used to diagnose leukopenia | ICD-10 | Japanese claims data Disease Code |
| --- | --- | --- |
| Leukopenia | D70 | 2880005 |
| Neutropenia | D70 | 2880001 |
| Febrile neutropenia | D70 | 8842350 |
| Idiopathic neutropenia | D70 | 8838180 |
| Granulocyte depletion | D70 | 2880009 |
| Drug-induced granulocytes | D70 | 8840703 |
| Agranulocytosis | D70 | 2880006 |
| pancytopenia | D169 | 2899005 |
| Aplastic anemia | D169 | 2849003 |

**Supplementary Table 3. Number of patients by type of G-CSF administration**

| G-CSF types | n | Percentage (%) |
| --- | --- | --- |
| Filgrastim | 30 | 79.0 |
| Lenograstim | 7 | 18.4 |
| Pegfilgrastim | 1 | 2.6 |
| total | 38 | 100.0 |

**Supplementary Figure 1. Study design**

* Patients who were prescribed an ATD during the 6 months prior to the observation period (April to September 2014) were excluded.

† Patients with disease codes for lung cancer, breast cancer, lymphoma, and leukemia were excluded.
